# Vesicovaginal fistula repair: comparative analysis of perioperative outcomes and predictors of success in open, laparoscopic, and robotic approaches

**DOI:** 10.1186/s40001-026-03937-5

**Published:** 2026-01-29

**Authors:** Zeyuan Wang, Gaurab Pokhrel, Shuanbao Yu, Haoke Zheng, Jin Tao, Yafeng Fan, Yunlong Liu, Jinjin Feng, Biao Dong, Tengfei Li, Xuanyi Ren, Xuepei Zhang

**Affiliations:** 1 Department of Urology, The First Affiliated Hospital of Zhengzhou University, No. 1 Jianshe East Road, Zhengzhou, 450052 China; 2 Department of Urology, Kaifeng Central Hospital, Kaifeng, China

## Abstract

**Background:**

To compare perioperative outcomes and evaluate predictors of successful repair in vesicovaginal fistula (VVF) cases among open, laparoscopic, and robotic-assisted approaches.

**Methods:**

This retrospective cohort study included 78 patients who underwent transabdominal VVF repair between December 2015 and July 2024 at a tertiary referral center. Patients were categorized based on surgical approach: open (*n* = 31), laparoscopic (*n* = 31), or robotic-assisted (*n* = 16). Data collected included demographics, fistula etiology and location, prior radiation exposure, operative time, blood loss, use of interposition flaps, and 3-month postoperative closure status.

**Results:**

Minimally invasive techniques (laparoscopic and robotic) demonstrated reduced operative time (*P* = 0.014) and blood loss (*P* < 0.001) compared to open surgery. Time to repair (*P* = 0.001) and previous repair attempts (*P* = 0.007) varied significantly across approaches. Post-hysterectomy fistulas had the highest success rate (95%), while radiation-induced (*P* = 0.031) and trigonal fistulas (failure rate 32% vs. 6% in non-trigonal; *P* = 0.004) were associated with poorer outcomes. Use of interposition flaps was more frequent in open cases but did not independently predict success. The surgical approach was not an independent predictor of closure rates; however, minimally invasive techniques were associated with better perioperative outcomes.

**Conclusions:**

This study highlights the importance of selecting the surgical approach based on fistula characteristics and clinical context. Minimally invasive techniques provided perioperative advantages, whereas open surgery remains essential for complex cases. Careful preoperative planning and well-informed clinical decision-making are crucial to optimize management. Further prospective, multicenter studies are warranted to confirm these findings and guide standardized best practices.

## Background

Vesicovaginal fistula (VVF) is an abnormal communication between the bladder and vagina that causes involuntary urinary leakage [1]. This condition has significant impact on women’s physical, psychological, and social well-being [2]. The etiology of VVF varies geographically. In low and middle-income countries, obstetric trauma is the most common cause. In contrast, in developed countries, gynecological surgeries, such as hysterectomy, are the leading contributors [3, 4]. Surgical repair remains the primary treatment, especially for complex, recurrent, or radiation-associated fistulas.

Surgical management of vesicovaginal fistulas has evolved from conventional open procedure to minimally invasive techniques including laparoscopic and robotic-assisted repair [5]. With favorable perioperative outcomes, including decreased operative times, reduced blood loss, and faster recovery, minimally invasive techniques are increasingly adopted to both simple and complex fistulas [6]. However, success rates remain variable, as outcomes are influenced by patient characteristics, fistula anatomy, prior interventions and surgeon expertise [7]. Identifying predictors of successful repair is essential for tailoring surgical strategies and improving patient outcomes.

Despite growing data on individual techniques, studies directly comparing perioperative outcomes and success rates of open and minimally invasive approaches within a single analysis remain limited. Moreover, the optimal surgical approach remains undefined, as outcomes are influenced by diverse clinical and anatomical factors [8]. In this single-center retrospective study of 78 patients, we evaluated perioperative outcomes and factors influencing the success of open, laparoscopic, and robotic-assisted vesicovaginal fistula repairs. These findings provide comparative, evidence-based data on common surgical approaches, supporting clinical decision-making and contributing to the expanding evidence on VVF research.

## Patients and methods

### Study design, setting and population

**Fig. 1:**
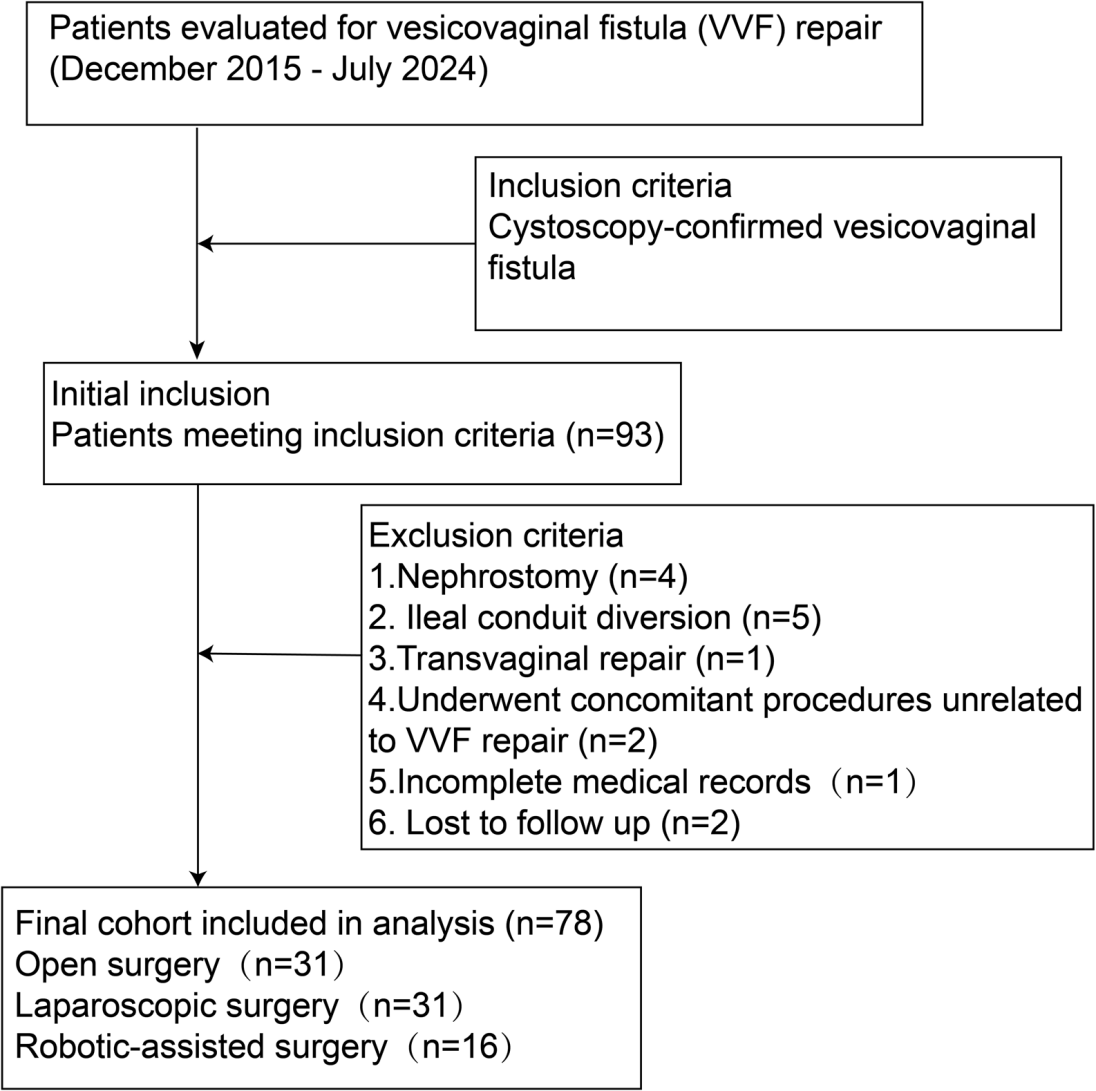
Patient selection and study cohort for vesicovaginal fistula repair

This single-center, retrospective cohort study was conducted at a university affiliated tertiary hospital. Data were collected from institutional review board-approved databases (2025-KY-1896). All surgeries were performed by a single experienced surgical team. The study included patients who underwent vesicovaginal fistula repair between December 2015 and July 2024. Laparoscopic repair was an established technique prior to the study period, and robotic-assisted surgery was introduced at our center in 2016. Exclusion criteria included incomplete medical records, loss to follow-up, vaginal repair and concomitant procedures unrelated to VVF repair. (Fig. 1).

### Data collection

Demographic, clinical, and surgical data were collected, including patient characteristics (age, body mass index (BMI), comorbidities, prior surgical history), fistula-specific factors (etiology, size, location, number), and surgical approach (open, laparoscopic, robotic), operative time, estimated blood loss (EBL), use of interposition tissue and intraoperative complications. Preoperative evaluation included history, physical exam, and cystoscopy to diagnose, locate, and size the fistula. Cross-sectional imaging (CT urogram/MRI) was used selectively for complex or recurrent cases to evaluate surrounding tissue and and rule out associated pathology. Follow-up data assessed patient-reported outcomes on success and failure and the presence of any urinary dysfunction at three months of surgery. Data accuracy was verified by two independent reviewers to ensure reliability and completeness.

### Surgical techniques

Surgery was performed using open, laparoscopic, or robotic-assisted approaches, with the choice of technique based on surgeon preference, fistula characteristics, and patient-specific factors. All procedures were conducted by a senior experienced surgeon via a transabdominal approach, adhering to established techniques [9–11].The choice of surgical approach and use of an interposition flap were determined by the senior surgeon through an integrated assessment of fistula characteristics (size, location, complexity, etiology), patient factors (body habitus, surgical/radiation history), and technical considerations (feasibility of minimally invasive surgery, resource availability). Open repair was generally preferred for complex cases, such as those involving radiation, large size, trigonal location, or extensive scarring. Interposition flaps were routinely employed, commonly in open repairs of complex fistulas.

#### Open repair

The bladder was accessed through a midline abdominal incision, adhesiolysis was done, and the fistula was exposed. Fibrotic tissue surrounding the fistula was excised, and the vaginal defect was repaired using continuous sutures followed by watertight bladder closure.

#### Laparoscopic repair

Standard laparoscopic instruments and techniques were used, with pneumoperitoneum created via a Veress needle and trocar placement. The fistula was exposed and excised, and the defect was repaired with continuous sutures.

#### Robotic-assisted repair

Following a similar transabdominal approach, robotic-assisted repairs were performed using the da Vinci Surgical System (Intuitive Surgical, Sunnyvale, CA). Pneumoperitoneum was created, trocars were placed, and the fistula was excised and repaired using continuous sutures.

**Fig. 2:**
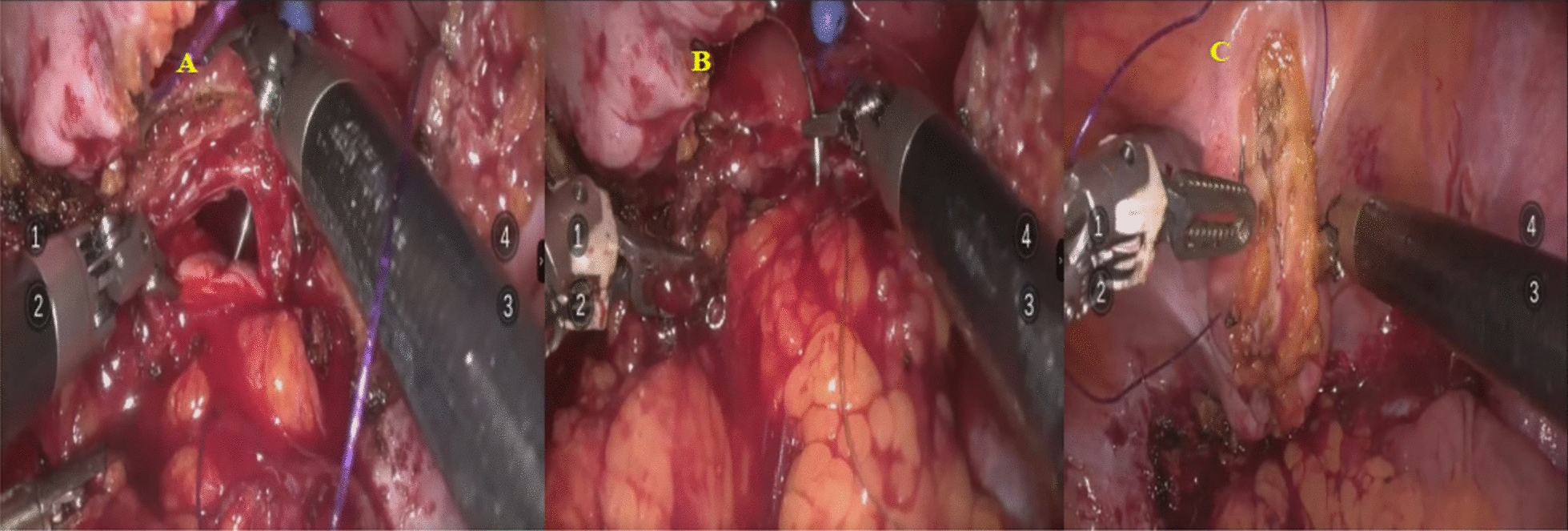
Intraoperative image during VVF repair, repair of the vaginal wall (**A**); flap interposition (**B**), bladder closure (**C**)

All repairs involved sharp excision of the fistulous tract with minimal cautery use, limited to devitalized or fibrotic tissue. Vascularized interposition grafts, such as omental flaps, were selectively applied based on fistula complexity, particularly in cases involving radiation-induced or recurrent fistulas. Intraoperative bladder filling was performed in all cases to test the integrity of the bladder closure. The bladder and vaginal defects were closed in separate layers using absorbable 3–0 polyglactin sutures. (Fig. 2).

A Foley catheter was placed in all patients postoperatively to maintain bladder drainage, typically for 10–21 days based on intraoperative findings. Routine postoperative cystography was not performed.

### Outcome and follow-up

The primary outcome was the success rate of VVF repair, defined as fistula closure with complete resolution of urinary leakage at three months, while longer-term clinical follow-up data was available for a subset of patients, it was not uniformly documented for all; therefore, the 3-month cystoscopic assessment served as the standardized endpoint for this comparative analysiswhile. Failure was defined as persistent fistula or recurrent urinary leakage. Secondary outcomes included operative time, blood loss, time to repair, interposition grafts and complication rates.

### Statistical analysis

Data were cleaned and analyzed using *R* software (version 4.3.3). Continuous clinical variables for VVF repair outcomes were summarized as median (interquartile range; IQR), while categorical variables were reported as percentages. Normality assumptions were assessed for continuous data: Student’s *t*-test was applied to normally distributed variables, and the Wilcoxon rank-sum test (Mann–Whitney U) was used for nonparametric comparisons. Categorical variables were analyzed using chi-square or Fisher’s exact tests, and Kruskal–Wallis rank sum test compared differences among the three surgical groups. Univariable logistic regression identified predictors of surgical success versus failure, with statistical significance defined as *α* = 0.05.

## Results

A total of 78 patients underwent VVF repair, including 31 open, 31 laparoscopic, and 16 robotic-assisted cases. The median age was 50 years (IQR: 44–54). The majority of fistulas (68%) were non-trigonal, and single fistulas were most prevalent (90%). Omental flaps were the most frequently used interposition tissue (60%).

### Perioperative factors

Significant differences were observed across surgical approaches for estimated blood loss (*P* < 0.001), operative time (*P* = 0.014), fistula etiology (*P* = 0.036), previous repair attempts (*P* = 0.007), and time to VVF repair (*P* < *0.001*). Robotic-assisted repairs demonstrated the shortest operative durations, laparoscopic approaches were associated with lower intraoperative blood loss, while open surgeries showed longer time to repair (Table 1).

**Table 1:** Patient demographics and perioperative outcomes

Parameters	Total	Open	Laparoscopic	Robotic	*P*
Age (year)	50(44–54)	50.5(44–53)	51(46–56)	49.5(44.75–54)	0.481
BMI(Kg/m^2^	24.3(22,26.8)	22.4(20.1,25.4)	24.9(22.1,27.4)	24.3(24.0,26.3)	0.203
DM (*n* %)					0.828
Yes	4(5)	2(6)	1(3)	1(6)	
No	74(95)	29(94)	30(97)	15(94)	
Radiotherapy (*n* %)					0.354
Yes	6(8)	4(13)	1(3)	1(6)	
No	72(92)	27(87)	30(97)	15(94)	
Time to repair (mon)	7(3–12)	9(6–36)	3(3–8)	7(3–12)	** < *0.001***
Etiology (*n* %)					***0.036***
Post hysterectomy	58(74)	18(58)	26(84)	14(88)	
Caesarean section	10(13)	7(23)	2(6)	1(6)	
Urological	5(6)	3(10)	1(3)	1(6)	
Others	5(6)	3(10)	2(6)		
Previous repair (*n* %)					***0.007***
Single	57(73)	17(55)	28(90)	12(75)	
Multiple	21(27)	14(45)	3(10)	4(25)	
Size (cm)	0.8(0.5–1.5)	0.9(0.5–2.1)	0.8(0.5–1.0)	1.0(0.55–1.25)	0.644
Location (*n* %)					0.026
Non-trigonal	53(68)	16(52)	26(84)	11(69)	
Trigonal	25(32)	15(48)	5(16)	5(31)	
Number (*n* %)					0.670
Single	70(90)	27(87)	29(94)	14(88)	
Multiple	8(10)	4(13)	2(6)	2(13)	
Interposition tissue (*n* %)					0.166
Omentum	47(60)	24(77)	15(48)	8(50)	
Rotational bladder flap	5 (6)	1(3)	2(6)	2(13)	
Fat tissue	6 (8)	3(10)	3(10)	0	
Others	20 (26)	3(10)	11(35)	6(38)	
Estimated blood loss (ml)	50(20–100)	100(50–100)	20(20–50)	25(20–50)	** < *0.00***1
Operative time (min)	111.5(83.25–142)	120(107.5–168.5)	100(76.5–133.5)	94(79–123.5)	***0.014***
Hospital stay (day)	7(7–9)	8(7–10.5)	7(6.5–8)	7(5–8.25)	0.170
Result (*n* %)					0.522
Success	67(86)	25(81)	28(90)	14(88)	
Failure	11(14)	6(19)	3(10)	2(13)	

Bold values indicates the specific *P*-values that are statistically significant (*P* 0.05)

### Predictors of successful repair

**Fig. 3:**
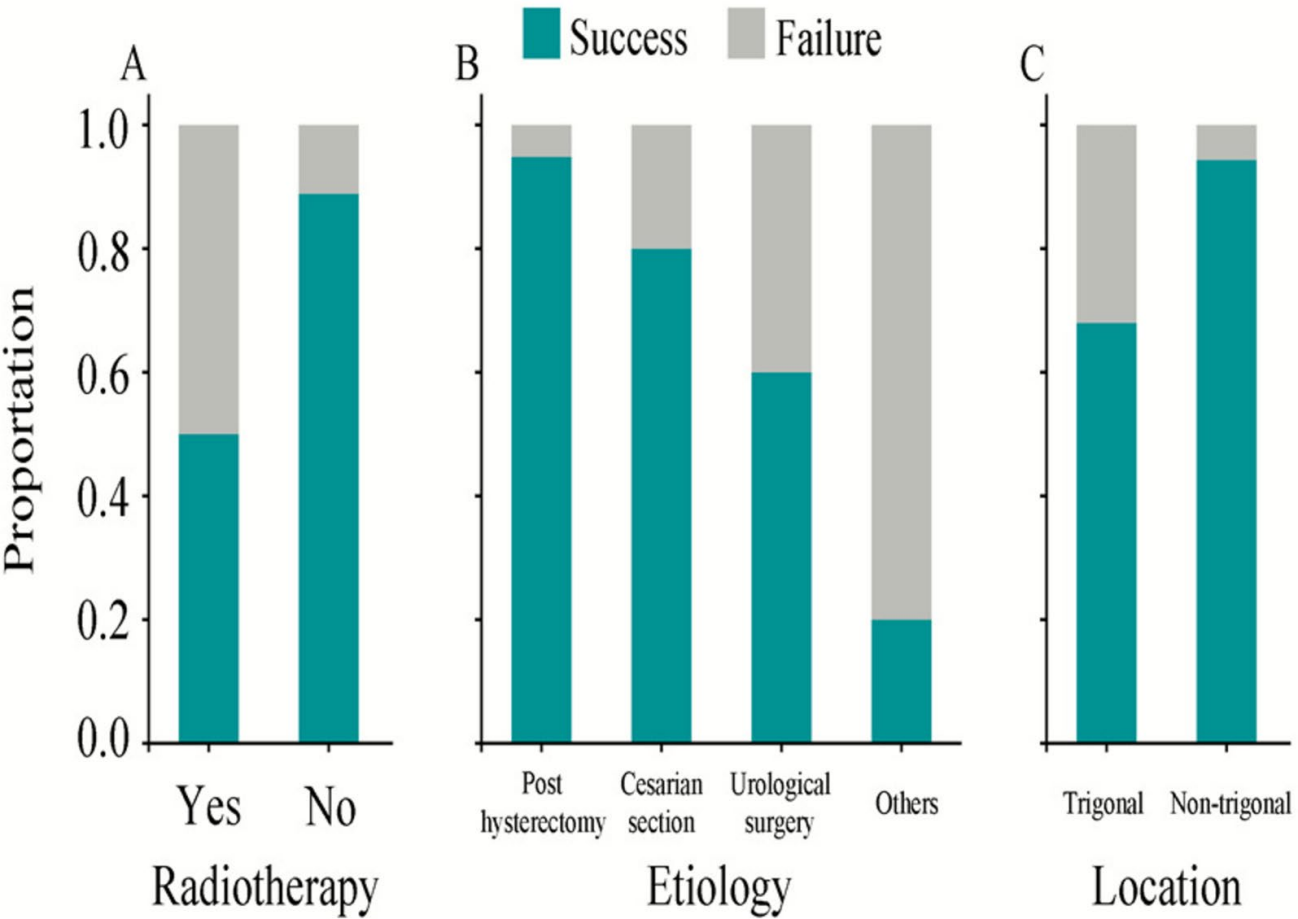
Factors associated with repair outcomes in VVF repair

Key predictors of surgical success included fistula etiology (*P* < 0.001), prior radiotherapy (*P* = 0.031), and anatomical location (*P* = 0.004) (Fig. 3) Post-hysterectomy fistulas demonstrated the highest success rates (95%), whereas radiation-associated cases and trigonal fistulas exhibited significantly poorer outcomes, with failure rates of 50% and 32%, respectively. Non-trigonal fistulas, in contrast, achieved a 94% success rate. (Table 2). Univariable logistic regression analysis showed that fistula etiology, prior radiotherapy (*P* = 0.021), and anatomical location (*P* = 0.005) were predictors of successful vesicovaginal fistula repair.

**Table 2:** Predictor of Successful vesicovaginal fistula repair

Parameters	Successful repair (*n* = 67)	Failure(*n* = 11)	*P*
Age (yr)	50(44.5, 54)	52(35, 58)	0.698
BMI(Kg/m2)	24.8(22.2,26.9)	22.4(20.8,23.6)	0.119
DM(n %)			0.093
Yes	2(3)	2(18)	
No	65(97)	9(82)	
Radiotherapy (*n* %)			***0.031***
Yes	3(4)	3(27)	
No	64(96)	8(73)	
Time to VVF repair (mon)	6(3–12)	8(4–20)	0.330
Etiology (*n* %)			** < *0.001***
Post hysterectomy	55(82)	3(27)	
Cesarian section	8(12)	2(18)	
Urological surgery	3(4)	2(18)	
Others	1(1)	4(36)	
Presentation (*n* %)			0.718
Primary	48(72)	9(82)	
Recurrent	19(28)	2(18)	
Fistula size (cm)	0.8(0.5–1.5)	1.0(0.6–2.5)	0.152
Location (*n* %)			***0.004***
Trigonal	17(25)	8(73)	
Non-trigonal	50(75)	3(27)	
Number of fistula (*n* %)			0.891
Single	60(90)	10(91)	
Multiple	7(10)	1(9)	
Interposition tissue (*n* %)			0.314
Omentum	41(61)	6(55)	
Rotational bladder flap	3(4)	2(18)	
Fat tissue	6(9)	0	
None	17(25)	3(27)	
Surgical approach (*n* %)			0.594
Robotic	14(21)	2(18)	
Laparoscopic	28(42)	3(27)	
Open	25(37)	6(55)	
Blood loss (ml)	50(20–75)	50(40–100)	0.093
Operative time (min)	110(81–142)	115(102.5–162.5)	0.282
Hospital stay (day)	7(6–9)	8(7–10)	0.177

*BMI,* Body Mass Index; *DM,* Diabetes mellitus; *VVF,* Vesicovaginal Fistula

Bold values indicates the specific *P*-values that are statistically significant (*P* 0.05)

### Surgical outcomes

Overall success, defined by fistula closure and resolution of symptoms at 3 months, was achieved in 67 of 78 cases (86%). Success rates by surgical approach were 81% in open, 90% in laparoscopic, and 88% in robotic-assisted repairs. Failure rates were higher in radiation-associated and trigonal fistulas, with open repairs primarily utilized in complex cases.

## Discussion

This study provides insight into the comparative performance of open, laparoscopic, and robotic-assisted vesicovaginal fistula repairs by evaluating perioperative outcomes along with key prognostic factors. By examining all three approaches within a single cohort, it adds perspective to the existing literature and contributes to the growing body of knowledge guiding evidence-based surgical decision-making.

The comparative analysis of perioperative outcomes across open, laparoscopic, and robotic-assisted VVF repairs demonstrates the relationship between surgical approach, case complexity, and procedural efficiency.

Robotic-assisted procedures were associated with the shortest median operative times and minimal blood loss, aligning with established advantages of enhanced visualization and instrument precision for delicate dissection [12]. It is important to note, however, that this finding is observed within a smaller cohort (*n* = 16) and may reflect selection bias toward anatomically favorable cases for this approach. Laparoscopic repairs demonstrated similarly low blood loss but required longer operative times, possibly reflecting technical limitations and a steeper learning curve. However, laparoscopic surgery remains the most commonly used minimally invasive approach due to its broader availability, lower cost, and growing surgeon proficiency [13]. The superior perioperative outcomes observed in both minimally invasive groups must therefore be interpreted within the context of this inherent selection bias, as these approaches were typically employed for favorable anatomy. Open surgery, while associated with greater blood loss and longer operative times, was the predominant approach for cases of higher complexity such as those with prior radiation, recurrence, where extensive dissection and the use of vascularized flaps are often necessary [14].

The significant variation in the use of interposition flaps across approaches further underscores differential case selection. The high utilization rate in the open group reflects its application in complex, high-risk scenarios where tissue reinforcement is routinely necessary. The comparatively lower but substantial use in minimally invasive groups (48% laparoscopic, 50% robotic), suggests that while precision techniques may reduce the universal need for flaps, they are still frequently employed based on intraoperative assessment [15].

The significant variation in time to repair across surgical approaches underscores the importance of case complexity and institutional resources. Open repairs experienced the longest delays (median: 9 months), often due to the need for preoperative optimization in high-risk cases, such as those involving radiation exposure or recurrence. In contrast, laparoscopic and robotic repairs, were performed earlier, with median intervals of 3 and 7 months, respectively [16, 17]. Although robotic surgery offers favorable perioperative outcomes, its broader application remains limited by factors such as cost, equipment availability, and the requirement for specialized surgical expertise, which may contribute to longer referral and scheduling intervals in many institutions. The prolonged time to repair observed in the open group indicates for its higher case complexity. Consequently, direct comparisons of outcomes between surgical approaches should be interpreted with appropriate caution, acknowledging this inherent selection bias.

These findings collectively suggest that minimally invasive techniques effectively reduce perioperative morbidity, while open surgery remains essential for anatomically complex or radiation-associated fistulas [11, 18]. Laparoscopic repair represents a commonly used and effective modality, offering favorable clinical outcomes while maintaining technical feasibility across varied surgical environments. Robotic surgery, despite its superior efficiency and precision, is often restricted to specialized centers due to limited accessibility and high costs. This underscores the need for individualized surgical planning, integrating surgical expertise, procedural complexity, and institutional resources to optimize outcomes [19].

The success of vesicovaginal fistula repair is governed by a complex interplay of patient-specific, anatomical, and technical factors. In this study, the overall success rate of 86% is consistent with previously reported outcomes, reflecting variability in fistula complexity and surgical expertise [20]. While laparoscopic (90%) and robotic-assisted (88%) repairs showed higher success rates than open surgery (81%), the differences was not statistically significant, likely due to the smaller robotic cohort and a higher proportion of complex cases treated with open group.

Fistula etiology, anatomical location and prior radiation exposure were important determinants of surgical outcomes (Fig. 3). Post-hysterectomy fistulas, commonly occurring in anatomically preserved and well-vascularized tissue planes, achieved the highest success rate (95%). In contrast, radiation-induced fistulas, typically characterized by suboptimal tissue conditions and fibrosis, were more prone to repair failure [21]. Interposition flaps may enhance tissue healing and reduce the risk of recurrence in these complex cases [22, 23]. Anatomical complexity was associated with lower success rates; trigonal fistulas located near important anatomical structure demonstrated a high failure rate compared to non-trigonal fistulas (*P* = 0.004). Interestingly, while trigonal location was a strong predictor of failure in our cohort, there was a clinical tendency to select open repair for these cases. This difference reflects the technical challenges of achieving a tight closure while preserving urinary tract function [24].

Interposition flaps are commonly used in complex or recurrent fistulas, particularly those associated with radiation exposure or large defects. While interposition grafts did not universally improve outcomes, their selective use in complex cases, including radiation-associated or large fistulas, may enhance local tissue healing and repair success [25]. Minimally invasive techniques, which demonstrated comparable success with reduced reliance on grafts, suggest that surgical precision and preservation of healthy tissue may minimize the need for reinforcement in appropriately selected patients.

While previous studies have proposed associations between number and size of fistula and repair success, the evidence remains heterogeneous and conflicting [26, 27]. In this study, these factors were not associated with successful outcomes. Similarly, intraoperative use of interposition flaps which is commonly advocated for complex or recurrent fistula did not correlate with improved success rates in our cohort, challenging assumptions about their universal utility [28]. Furthermore, surgical modality (open vs. minimally invasive) did not independently predict outcomes, despite perioperative advantages observed with laparoscopic and robotic techniques. Despite growing interest in minimally invasive techniques, existing evidence remains insufficient to establish their superiority over traditional open surgery [29].

These findings suggests that individualized surgical planning is essential. Minimally invasive techniques offer perioperative advantages for selected cases, such as post-hysterectomy fistulas, whereas open surgery remains fundamental for managing complex scenarios like radiation-induced defects. Expanding access to laparoscopic training and investing in robotic platforms could extend the benefits of minimally invasive surgery to more clinical settings [30].

## Limitations

This study has several important limitations. Its retrospective, single-center design introduces unavoidable selection bias, as the choice of surgical approach was influenced by surgeon preference, evolving surgeon experience, referral patterns, evolving institutional practice, and fistula characteristics rather than randomization. The sample size, particularly for the robotic cohort, may underpower the detection of clinically meaningful differences in success rates. Generalizability may be limited by the homogeneous patient population and the lack of standardized protocols for elements like interposition flap use. Future prospective, multicenter studies with a long-term follow-up period are needed to validate these findings and refine evidence-based guidelines.

## Conclusions

This study identifies key differences in perioperative outcomes and determinants of surgical success in VVF repair. The selection of surgical approach, guided by fistula etiology, anatomical complexity, and clinical context is critical for optimizing outcomes. Minimally invasive techniques offer clear perioperative advantages, while open surgery remains essential for complex cases. These findings underscore the need for tailored surgical planning to enhance decision-making and patient outcomes. Future multicenter prospective studies with standardized protocols are necessary to validate these findings and inform future clinical practices.

## Data Availability

The datasets used and/or analyzed during the current study are available from the corresponding author on reasonable request.

## Abbreviations

VVF: Vesicovaginal Fistula
EBL: Estimated Blood Loss
IQR: Interquartile Range
BMI: Body Mass Index
